# Death of District Physician Vaughn

**Published:** 1891-04

**Authors:** 


Death of District-Physician Vaughn. — Dr. Frank Owen Vaughn died recently at his home, No. 247 Swan street. He was forty-four years old, and had lived here all his life. His death resulted from typhoid fever. He graduated from the medical department of the University of Buffalo in 1880, and began the practice of his profession on Swan street. A year later he was appointed a district physician and held the position until his death. He leaves a wife and daughter, and one sister—Mrs. Stewart Elder of this city. He was a Mason of high standing, a member of the Masonic Acacia club, and was also a member of the A. O. U. W., the E. O. M. A., the K. of H., the Erie County Medical Society, and the State Medical Association. Dr. Vaughn was held in high esteem by all who knew him. The funeral took place Sunday afternoon, and the interment was at Forest Lawn. The Erie County Medical Society held a special meeting, March 20, 1891, and passed the following:

WHEREAS, Frank O. Vaughn, a member of our Society, and a graduate of the Medical Department of the University of Buffalo, 1880, and since actively engaged in the calling of his profession, succumbed to its arduous duties on the 18th day of March, 1891, therefore be it

*Resolved,* That we greatly mourn the loss of our late brother; that we lose in him an earnest and active member; a man of noble character, of candid and earnest demeanor, and a friend to humanity.

*Resolved,* That we tender to the afflicted family of our deceased brother our heartfelt sympathy in this, their hour of great bereavement.

*Resolved,* That we will attend his funeral in a body.

Copies of these resolutions to be spread on the minutes of the Society and sent to his family.

F. W. ABBOTT,<br>JOHN J. WALSH,<br>J. G. MEIDENBAUER, } Committee.

---

DR. FRANK P. FOSTER, the able editor of the *New York Medical Journal,* has been appointed Librarian of the New York Hospital Library. No better man could have been selected. We congratulate the library accordingly.—*Medical Record.*

